# Characterization of the complete mitochondrial genome of band-rumped Storm-petrel, *Hydrobates castro*

**DOI:** 10.1080/23802359.2018.1501320

**Published:** 2018-10-31

**Authors:** Jin-Qing Jiang, Qing-Hua Wang, Tian-Zeng Gao

**Affiliations:** a College of Animal Science and Veterinary Medicine, Henan Institute of Science and Technology, Xinxiang, China;; b Henan Guangan Biotechnology Limited Company, Zhengzhou, China

**Keywords:** Band-rumped Storm-petrel, *Hydrobates castro*, mitochondrial genome, phylogenetic analysis

## Abstract

In this study, the complete mitochondrial genome of band-rumped Storm-petrel, *Hydrobates castro,* was determined through sequencing of PCR fragments. The complete mitochondrial genome of *H. castro* was 17,065 bp in length and encoded 13 protein-coding genes, 22 transfer RNA (tRNA) genes, and two ribosomal RNA genes. The overall nucleotide composition is: 30.4% A, 24.5% T, 30.9% C, and 14.2% G, with a total G + C content of 45.1%. By phylogenetic analysis using Bayes method, *H. castro* showed the closest relationship with the white-faced storm petrel (*Pelagodroma marina*).

The Band-rumped Storm-Petrel, which is known as Storm-Petrel in Europe, is found both in the Pacific and Atlantic oceans. This marine species is highly pelagic, occurring in warm waters and rarely approaching land except near colonies. It feeds mostly on planktonic crustaceans, fish and squid but will also feed on human refuse. It mainly feeds in the day on the wing by pattering, dipping and also by surface-seizing. Its breeding season varies locally in colonies on undisturbed islets, in flat areas near the sea or inland on cliffs (BirdLife International 2016).

In this study, the feet of three dead adults were collected at Azores, Portugal (38°60′N, 28°80′W) and stored in lysis buffer. The total DNA was extracted using the commercial Animal Tissues Genomic DNA Extraction Kit (Solarbio, Beijing, China) following the manufacturer’s instructions, and then used as the template for polymerase chain reaction (PCR) amplifications. Both tissue and extracted genomic DNA was stored in the Zoology Comprehensive Laboratory from College of Animal Science and Veterinary Medicine. The amplified products were sequenced using the amplification primers. All sequencing was done by ABI3730. Some small number of PCR products that could process complex secondary structures or high A + T content, were cloned into the PMD-19T vector (TaKaRa, Dalian, China), then transformed to JM109 competent cell (TaKaRa, Dalian, China) for sequencing. All sequencing sequences were assembled with the program Seqman in the DNASTAR package (Burland 1999).

Protein coding genes were predicted using the MITOS web server (Bernt et al. 2013) using the vertebrate mitochondrial genetic code. Protein-coding regions and ribosomal RNA genes were also annotated manually and confirmed by comparison to the mitogenome of *Pelagodroma marina* (GenBank accession: KC875856.1) that available in GenBank. The transfer RNA (tRNA) genes were identified and assigned putative secondary structures using the program tRNAscan-SE (Lowe et al. 1997) or by manually identifying potential secondary structures and anticodon sequences through visual inspection. The graphical map of the complete mitochondrial genome was drawn using the online software OrganellarGenomeDRAW (Lohse et al. 2007).

The complete mitogenome of *Hydrobates castro* (GenBank accession: MH433599) is a closed-circular molecule of 17,065 bp in length, which is a litter longer than other sequenced *Microtus* mitogenomes. It presents the typical set of 37 genes observed in metazoan mitogenomes, including 13 PCGs (*cox*1-3, *co*b, nad1-6, *nad*4L, *atp*6, and *atp*8), 22 tRNA genes (one for each amino acid, two each for leucine and serine), two genes for ribosomal RNA subunits (*rrn*S and *rrn*L). The gene arrangement of this mitochondrial genome was the same as that of *Pelagodroma marina*. Genome organization of *H. castro* is very compact, encoding 16,612 bp functional regions (including D-loop region).

For phylogenetic analysis assessing the relationship of this mitogenome, we selected other nine related Neognathae mitogenomes downloaded from GenBank. The genome-wide alignment of all mt genomes was done by HomBlocks (Bi et al. 2018), resulting in 11,100 positions in total, including almost all whole or partial PCGs and rRNA genes. The whole genome alignment was analysed by PhyloBayes ver. 3.3 (Lartillot et al. 2012) under the CAT-GTR + Γ model. Two independent MCMC analyses were run for 10,000 cycles in PhyloBayes. Convergence was checked based on time-series plots of the likelihood scores using Tracer (http://tree.bio.ed.ac.uk/software/tracer/). The first 5000 cycles were discarded as burn-in, and the remaining trees were summarized to obtain Bayesian posterior probabilities (BPPs). The resulting tree was represented and edited using FigTree v1.4.1 (http://www.umiacs.umd.edu/∼morariu/figtree/). As shown in Figure 1, the phylogenetic positions of these 10 mt genomes were successfully resolved with full BPPs supports except one node. As expected, *H. castro* showed the closest relationship with the white-faced storm petrel (*Pelagodroma marina*).

**Figure 1.**
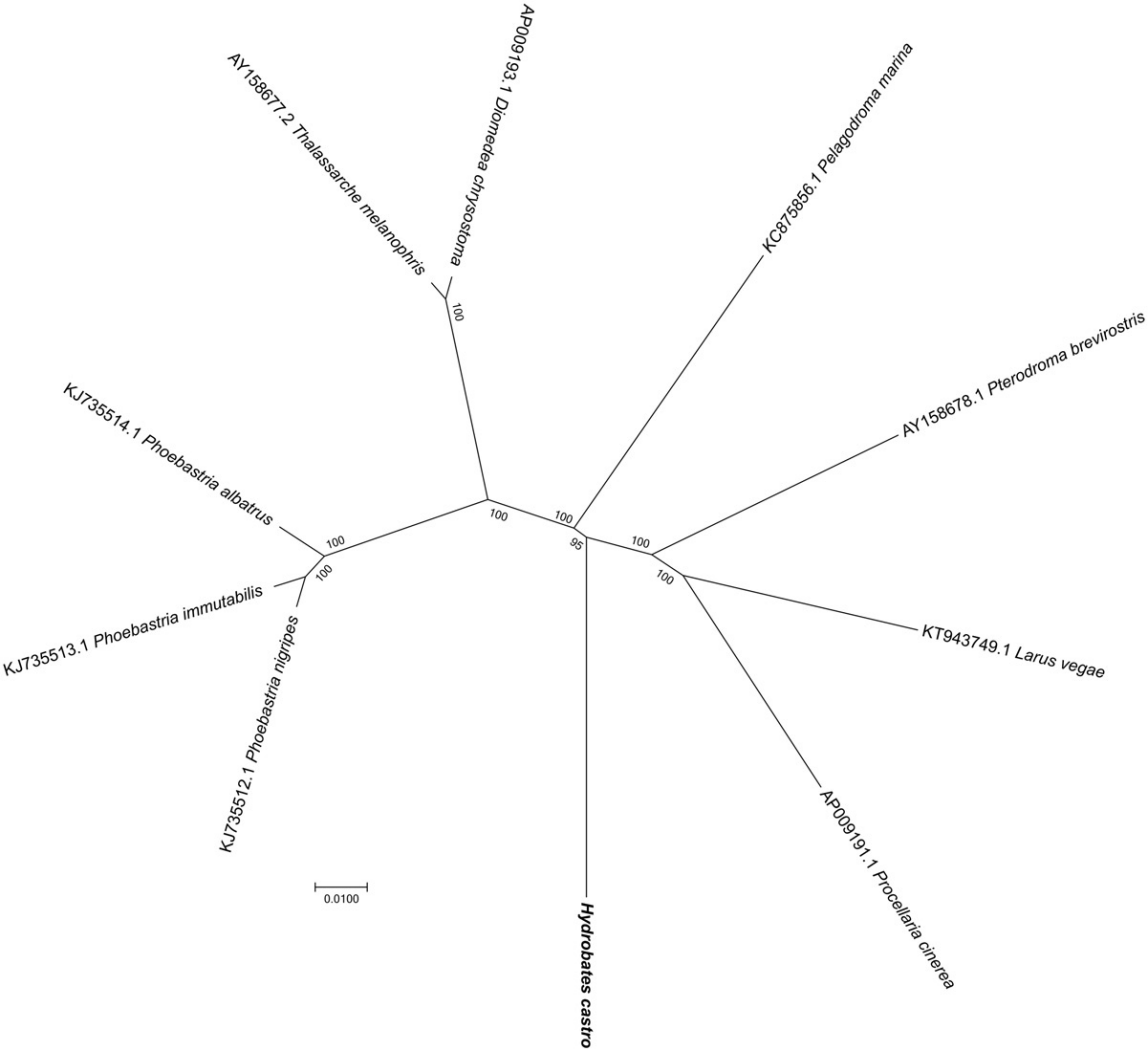
Phylogenetic tree yielded by Bayesian analysis of 10 fabids Neognathae genomes. Bayesian consensus tree is shown with support indicated by numbers at branches, representing percentage of Bayesian posterior probabilities (BPPs).
